# A rational-emotive stress management intervention for reducing job burnout and dysfunctional distress among special education teachers: An effect study: Erratum

**DOI:** 10.1097/MD.0000000000010827

**Published:** 2018-05-18

In the article, “A rational-emotive stress management intervention for reducing job burnout and dysfunctional distress among special education teachers: An effect study”^[1]^, which appeared in Volume 97, Issue 17 of *Medicine*, Dr. Eucharia A. Onu's name appeared incorrectly as Eucharia U. Onu.
